# Supplementary material for: Clinically actionable pharmacogenomic landscape of antidepressants and antipsychotics in Qatar: a population-based cohort study
Source: Camb Prism Precis Med. 2025 Apr 28;3:e4. doi: 10.1017/pcm.2025.2 (PMC12277200; doi:10.1017/pcm.2025.2)
Supplement: Velayutham et al. supplementary material 2 — Velayutham et al. supplementary material [file S275261432500002Xsup002.pdf]

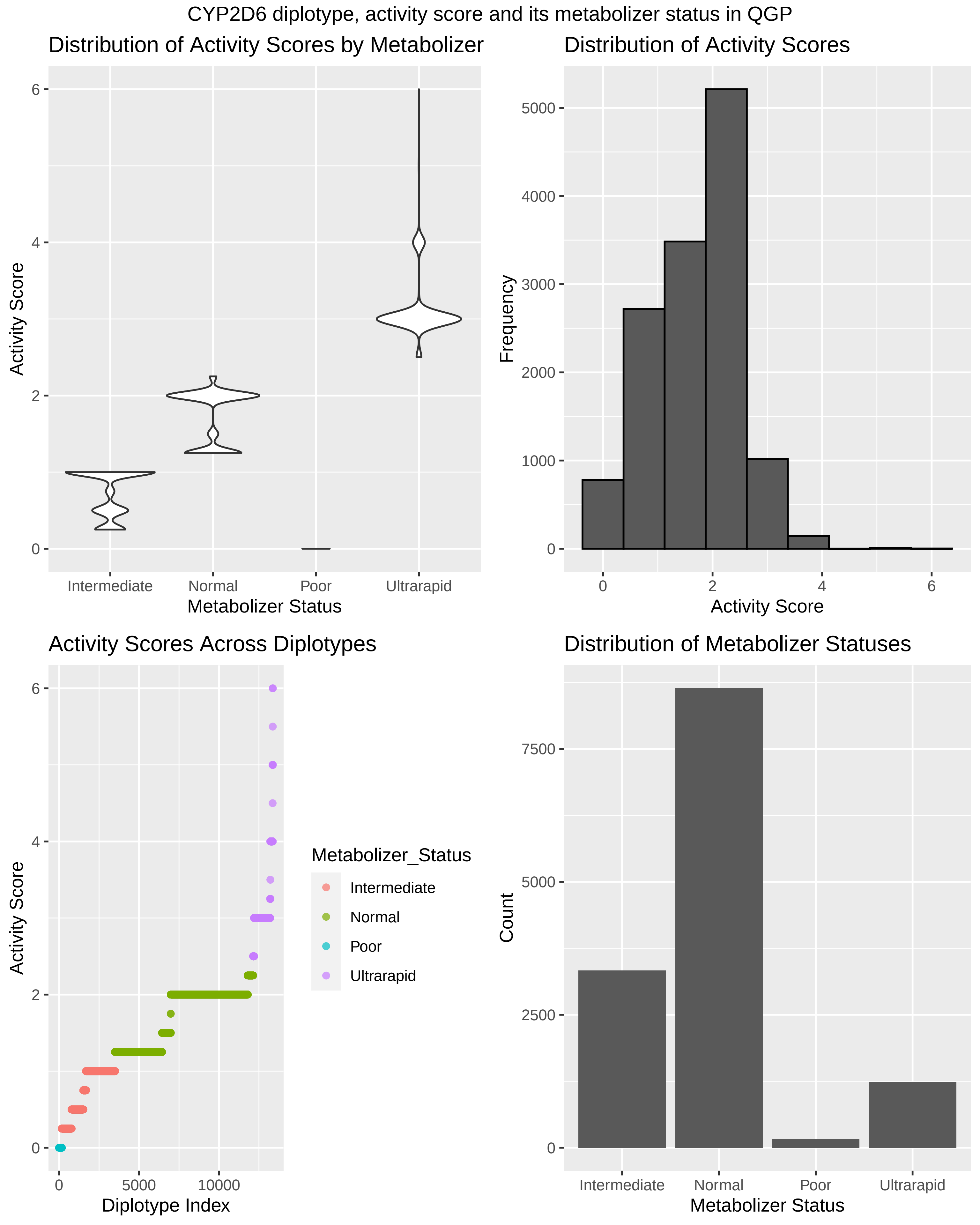

CYP2D6 diplotype, activity score and its metabolizer status in QGP
Distribution of Activity Scores by Metabolizer
Activity Score
6
4
2
0
Intermediate
Normal
Poor
Ultrarapid
Metabolizer Status
Distribution of Activity Scores
Frequency
5000
4000
3000
2000
1000
0
0
2
4
6
Activity Score
Activity Scores Across Diplotypes
Activity Score
6
4
2
0
0
5000
10000
Diplotype Index
Metabolizer_Status
Intermediate
Normal
Poor
Ultrarapid
Distribution of Metabolizer Statuses
Count
7500
5000
2500
0
Intermediate
Normal
Poor
Ultrarapid
Metabolizer Status
